# Intracellular translocation of HMGB1 is important for Zika virus replication in Huh7 cells

**DOI:** 10.1038/s41598-022-04955-z

**Published:** 2022-01-20

**Authors:** Kim-Ling Chin, Nurhafiza Zainal, Sing-Sin Sam, Pouya Hassandarvish, Rafidah Lani, Sazaly AbuBakar

**Affiliations:** 1 Tropical Infectious Diseases Research and Education Centre (TIDREC), Universiti Malaya, Kuala Lumpur, Malaysia; 2 Department of Medical Microbiology, Faculty of Medicine, Universiti Malaya, Kuala Lumpur, Malaysia

**Keywords:** Virology, Protein transport, Infectious diseases

## Abstract

Neonatal microcephaly and adult Guillain–Barré syndrome are severe complications of Zika virus (ZIKV) infection. The robustly induced inflammatory cytokine expressions in ZIKV-infected patients may constitute a hallmark for severe disease. In the present study, the potential role of high mobility group box 1 protein (HMGB1) in ZIKV infection was investigated. HMGB1 protein expression was determined by the enzyme-linked immunosorbent assay (ELISA) and immunoblot assay. HMGB1’s role in ZIKV infection was also explored using treatment with dexamethasone, an immunomodulatory drug, and HMGB1-knockdown (shHMGB1) Huh7 cells. Results showed that the Huh7 cells were highly susceptible to ZIKV infection. The infection was found to induce HMGB1 nuclear-to-cytoplasmic translocation, resulting in a > 99% increase in the cytosolic HMGB1 expression at 72-h post-infection (h.p.i). The extracellular HMGB1 level was elevated in a time- and multiplicity of infection (MOI)-dependent manner. Treatment of the ZIKV-infected cells with dexamethasone (150 µM) reduced HMGB1 extracellular release in a dose-dependent manner, with a maximum reduction of 71 ± 5.84% (P < 0.01). The treatment also reduced virus titers by over 83 ± 0.50% (P < 0.01). The antiviral effects, however, were not observed in the dexamethasone-treated shHMGB1 cells. These results suggest that translocation of HMGB1 occurred during ZIKV infection and inhibition of the translocation by dexamethasone coincided with a reduction in ZIKV replication. These findings highlight the potential of targeting the localization of HMGB1 in affecting ZIKV infection.

## Introduction

Zika virus (ZIKV) epidemics in the Pacific and Americas^1^ were declared as a Public Health Emergency of International Concern (PHEIC) in February 2016^2^. The infection was caused by ZIKV, an arthropod-borne virus (arbovirus) that belongs to the genus *Flavivirus*, family *Flaviviridae*. The ZIKV epidemic becomes a major concern as it is associated with severe neurological disorders, including microcephaly in newborns and Guillain–Barré syndrome in adults^3,4^. ZIKV has a wide tissue tropism and it has been found in the hemolymphatic, gastrointestinal, genitourinary, ophthalmic and reproductive systems^5–8^. ZIKV is known to also infect liver cells including the hepatocytes^9^. The infection induced liver inflammation or hepatitis which can result in liver damage and dysfunction in infected patients^10,11^. To date, there is no specific antiviral or vaccine available to treat or prevent ZIKV infection.

HMGB1 is a highly conserved DNA-binding protein and mainly resides in the nucleus, regulating the DNA transcription and nucleosome stability^12^. During infection and cell injury, acetylated HMGB1 is translocated from the nucleus to the extracellular milieu to act as a proinflammatory cytokine, causing inflammatory responses^13^ as seen in various virus-infected cells such as hepatitis C virus, dengue virus, West Nile virus, herpes simplex virus, human immunodeficiency virus and severe acute respiratory syndrome coronavirus‐2^14–20^. HMGB1 levels in dengue and hepatitis B infected patients’ sera were found to be positively correlated to viral load and disease symptoms^21,22^. Although the HMGB1 canonical pathway was found to be activated in both in vitro^23,24^ and ZIKV presymptomatic or asymptomatic patient plasma^25^, the role of HMGB1 in ZIKV infection remains largely unknown.

Dexamethasone is a glucocorticoid that has been broadly used due to its rapid anti-inflammatory and immunosuppressive impacts. The protective role of dexamethasone against various diseases, including sepsis^26^, severe acute pancreatitis^27^, acute lung injury^28^ through inhibiting HMGB1-mediated inflammation, has been proposed. Dexamethasone has been shown to inhibit HMGB1 nuclear-to-cytoplasmic translocation via acetyltransferase attenuation, hence reducing the HMGB1-TLR4 inflammatory pathway^29^. On the other hand, recent studies showed that dexamethasone possesses the antiviral potential against many viruses such as human rhinovirus^30^, foot-and-mouth disease virus^31^ and dengue virus^32^. The following study aimed to examine the dynamic of HMGB1 expression following ZIKV infection in vitro and its potential role in the pathogenesis of Zika with interest in the liver infection using the liver originated Huh7 hepatoma cells.

## Results

### ZIKV caused productive infection in Huh7 cells

**Figure 1:**
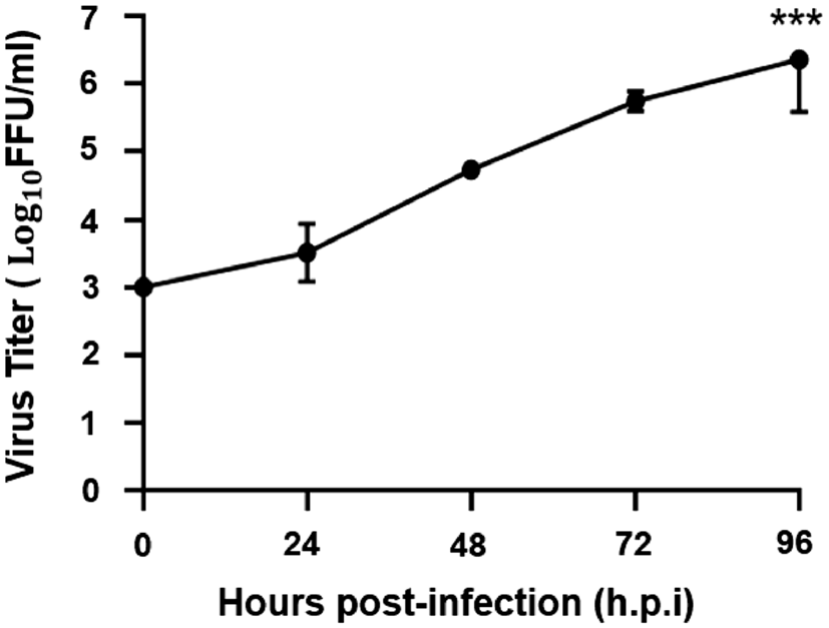
Replication kinetics of ZIKV in Huh7 cells. Huh7 cells were infected at a multiplicity of infection (MOI) of 1 in 96-well plate and the replication of virus was studied after 1–4 days post-infection (p.i). Focus-forming assay on Vero cells was performed to determine virus titers of infected cell culture supernatants. Asterisks (***) indicate a significant difference (P < 0.001).

The Huh7 cells were infected with the Asian ZIKV strain P6–740 at a multiplicity of infection (MOI) of 1 in a 96-well plate and virus replication was monitored at selected time points for 4 days post-infection. At 24, 48, 72 and 96 h post-infection (h.p.i), two hundred microliters (200 µL) of supernatant of the infected cell cultures was harvested for virus titration using the focus-forming assay as previously described^33^. A gradual increase in the production of infectious virus was observed over the infection period (Fig. 1). From a titer of 3 ± 0 log10 FFU/mL at 0 h.p.i, the ZIKV replicated to 3.511 ± 0.033 log10 FFU/mL at 24 h.p.i. (P > 1.0), 4.734 ± 0.010 log10 FFU/mL (1-log increase, P > 1.0) at 48 h.p.i, 5.744 ± 0.012 log10 FFU/mL (2-log increase, P > 0.1) at 72 h.p.i, and a maximum titer of 6.362 ± 0.061 log10 FFU/mL (3-log increase, P < 0.001) at 96 h.p.i (Fig. 1). These findings suggested that Huh7 cells supported ZIKV replication well.

### ZIKV infection induced translocation of nuclear HMGB1 into the cytosol

**Figure 2:**
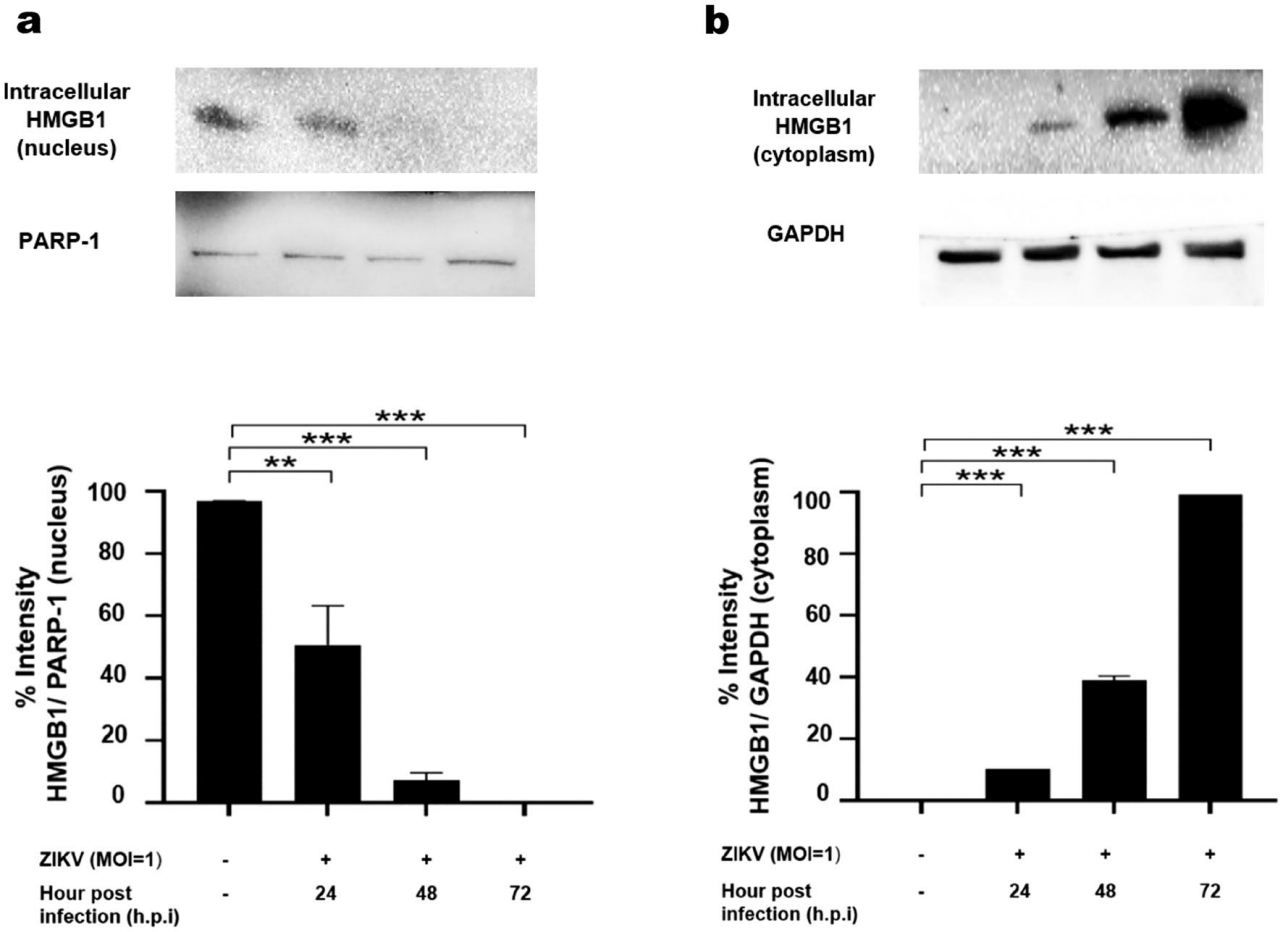
HMGB1 translocation during ZIKV infection. Huh7 cells were mock-infected or infected with ZIKV at an MOI of 1. Cell lysates were harvested at 24-, 48- and 72-h post-infection (h.p.i). The nuclear (**a**) and cytosolic (**b**) proteins were determined by immunoblot assay for HMGB1 detection. Statistical significances between the groups were indicated: *P < 0.05, **P < 0.01, ***P < 0.001.

The nuclear and cytosolic HMGB1 protein expressions of the mock-infected or ZIKV-infected Huh7 cells were determined using immunoblot assay. The nuclear and cytosolic HMGB1 expressions were normalized to the PARP-1 and GAPDH levels, respectively (Fig. 2). The HMGB1 was detected mainly in the nucleus of the mock-infected Huh7 cells after 72 h of the incubation period (Fig. 2). During ZIKV infection, the accumulation of nuclear HMGB1 was reduced to 51% at 24 h.p.i (P < 0.01), to 7.2% at 48 h.p.i (P < 0.001) and 0% at 72 h.p.i. (P < 0.001), in comparison to that of the mock-infected Huh7 cells (Fig. 2a). On the other hand, the ZIKV infection resulted in increased level of HMGB1 in the cytoplasm by 11% at 24 h.p.i (P < 0.001), 39% at 48 h.p.i (P < 0.001) and 99% at 72 h.p.i. (P < 0.001) as compared to the mock-infected cells (Fig. 2b). These results suggested that the ZIKV infection induced the translocation of HMGB1 from the nucleus to the cytoplasm in Huh7 cells.

### ZIKV infection induced HMGB1 release from Huh7 cells in a time- and MOI-dependent manner

**Figure 3:**
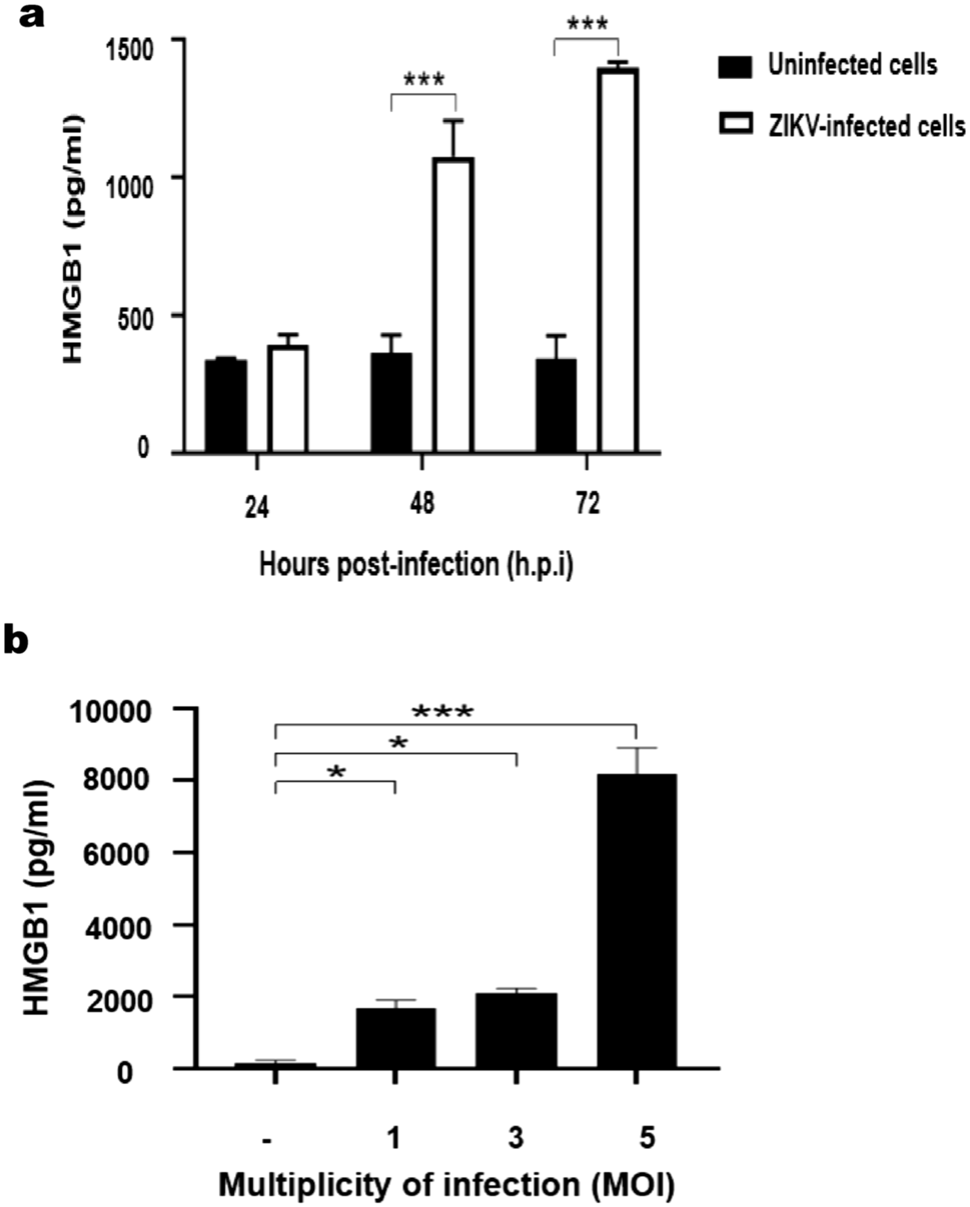
Extracellular HMGB1 levels during ZIKV infection: (**a**) Huh7 cells were mock-infected or infected with ZIKV at an MOI of 1. Supernatants of cell culture were harvested at 24-, 48- and 72-h post-infection (h.p.i) and the level of HMGB1 was measured by ELISA. Asterisks (**) indicate a significant difference (P < 0.01). (**b**) Huh7 cells were mock-infected or infected with ZIKV at an MOI of 1, 3 and 5. Supernatants were then harvested at 72 h.p.i for extracellular HMGB1 detection by ELISA. Statistical differences between the groups were indicated: *P < 0.05, **P < 0.01, ***P < 0.001.

The extracellular HMGB1 levels in the culture supernatant of mock-infected Huh7 cells remained constant over the 72 h incubation period, with an average value of 346.83 ± 20.19 pg/mL. In contrast, the ZIKV infection at MOI of 1 significantly increased the extracellular HMGB1 levels at 48 h.p.i (1074.75 ± 91.25 pg/mL, P < 0.001) and 72 h.p.i (1397.25 ± 13.75 pg/mL, P < 0.001), in comparison to those of the mock-infected cells (Fig. 3a). Our results also showed that the HMGB1 release from the infected cells was MOI-dependent. At 72 h.p.i, the levels of extracellular HMGB1 increased from 1671.25 ± 162.5 pg/mL (P < 0.05) at MOI of 1, to 2115 ± 81.25 pg/mL (P < 0.05) at MOI of 3 and 8165 ± 531.25 pg/mL (P < 0.001) at MOI of 5 (Fig. 3b). These results suggested that the ZIKV infection resulted in extracellular release of HMGB1 from Huh7 cells.

### Dexamethasone inhibited the release of extracellular HMGB1 and ZIKV replication in a dose-dependent manner

**Figure 4:**
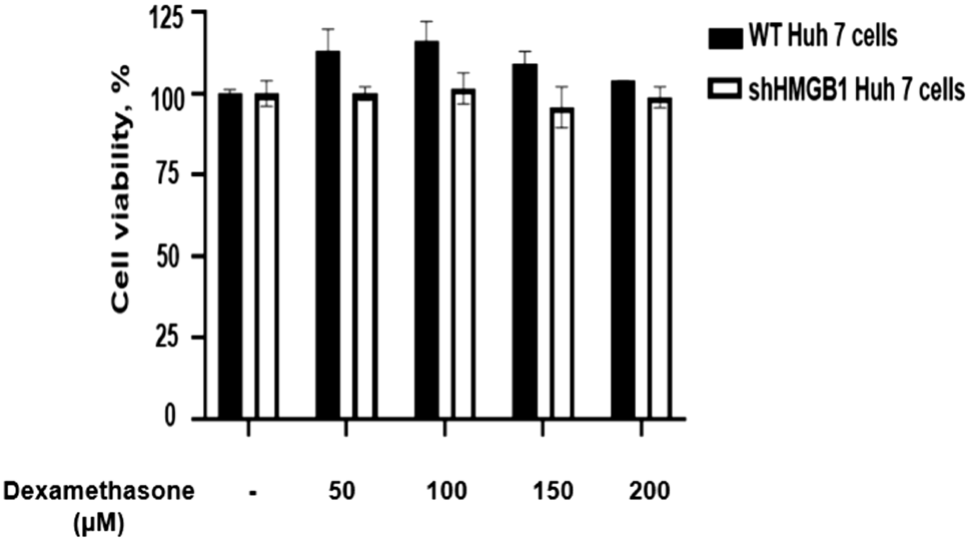
Cytotoxic effects of dexamethasone on wild-type (WT) and HMGB1-knockdown (shHMGB1) Huh7 cells. The cytotoxicity of dexamethasone was determined using the MTS assay. Both cells were treated with dexamethasone at concentrations of 0 μM, 50 μM, 100 μM, 150 μM and 200 μM for 72 h. The experiments were performed in triplicates and the data obtained were analyzed using Graph Pad Prism 8.

**Figure 5:**
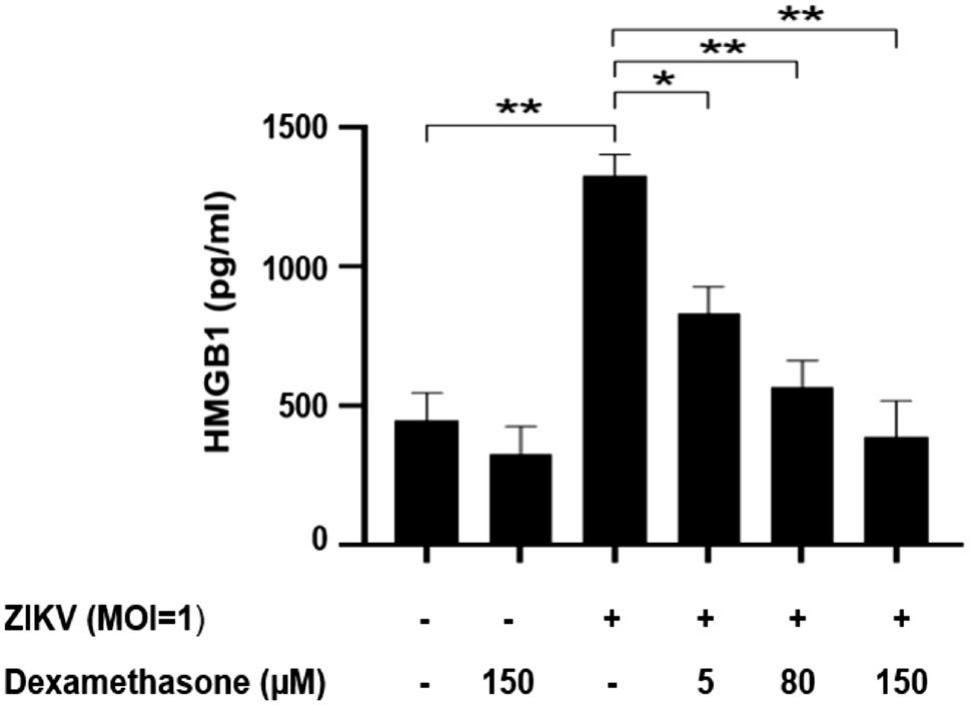
Effects of dexamethasone treatment on extracellular HMGB1 level during ZIKV infection. Huh7 cells were treated with various concentrations of dexamethasone (0, 5, 80, 150 μM) after ZIKV infection of ZIKV at MOI of 1. After 72 h, supernatants of cell culture were harvested and the level of HMGB1 was analyzed by ELISA. Significant differences between the groups were indicated: *P < 0.05 and **P < 0.01.

**Figure 6:**
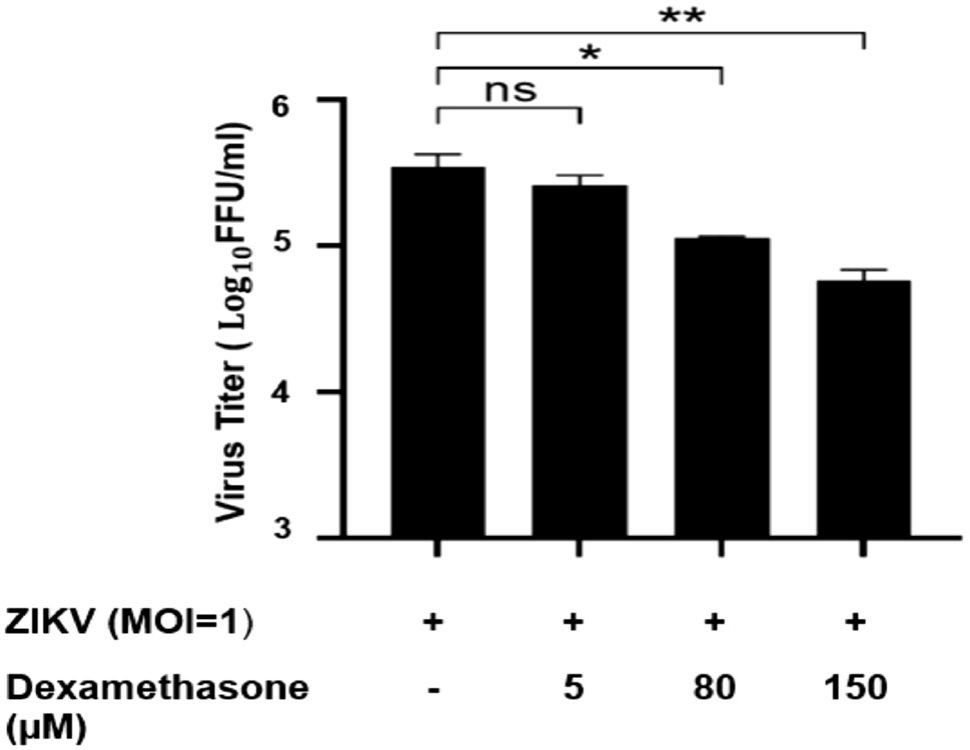
Inhibitory effects of dexamethasone against ZIKV replication. Wild-type (WT) Huh7 cells were treated with various concentrations of RESV (0, 5, 80, 150 μM) after ZIKV infection at MOI of 1. The cell supernatants were harvested at 72 h.p.i and virus titers were determined using the focus-forming assay. Statistical significances between the groups were indicated: *P < 0.05 and **P < 0.01.

Dexamethasone showed low cytotoxicity for Huh7 cells; almost 100% of the Huh7 remained viable following treatments with 50–200 µM dexamethasone (Fig. 4). After ZIKV infection at MOI of 1, the Huh7 cells were treated with 0, 5, 80 and 150 µM of dexamethasone and the HMGB1 release from the infected cell and virus titers were assessed at 72 h.p.i (Figs. 5, 6). The extracellular HMGB1 levels of ZIKV-infected cells were significantly reduced from 1326 ± 54 pg/mL for the mock treatment, to 829 ± 69 pg/mL (P < 0.05), 564 ± 68 pg/mL (P < 0.01) and 385 ± 93 pg/mL (P < 0.01) by treatment with dexamethasone at 5, 80 and 150 µM, respectively (Fig. 5). Furthermore, the treatment of ZIKV-infected Huh7 cells with 5 µM, 80 µM and 150 µM of dexamethasone decreased the infectious virus titers by 25% (P > 0.05), 67% (P < 0.05) and 83% (1 log, P < 0.01), respectively (Fig. 6), in comparison to the mock-treated control.

### Anti-ZIKV replication effects of dexamethasone were dependent on the presence of HMGB1

**Figure 7:**
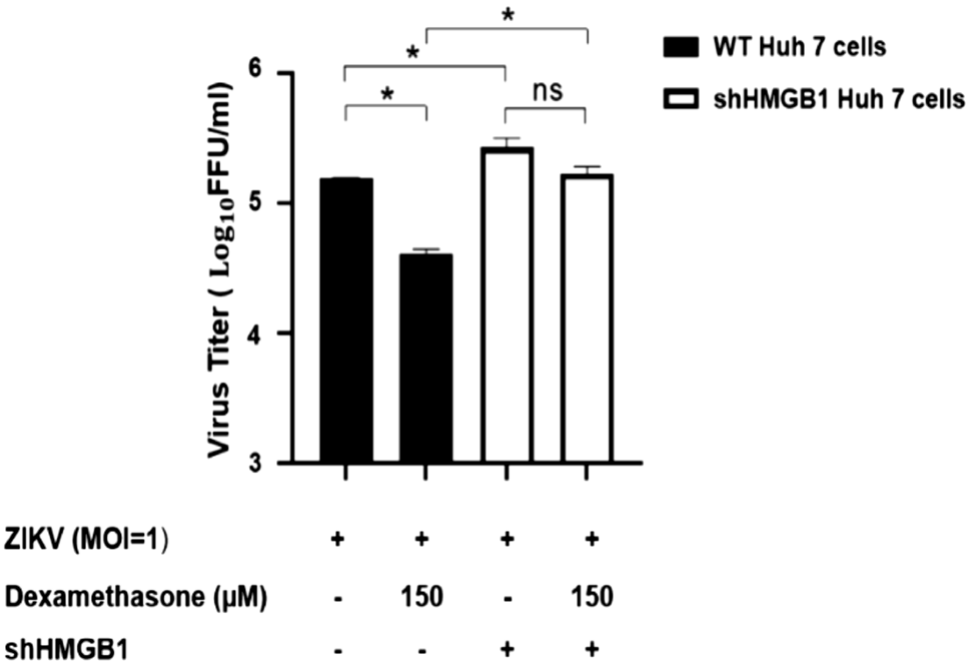
Role of HMGB1 in dexamethasone’s antiviral response against ZIKV. Wild-type (WT) and HMGB1-knockdown (shHMGB1) Huh7 cells were treated with 0 and 150 μM of RESV after ZIKV infection at MOI of 1. The cell supernatants were harvested at 72 h.p.i and virus titers were determined using the focus-forming assay. Asterisks (*) indicate a significant difference (P < 0.05).

The involvement of HMGB1 in the dexamethasone’s anti-ZIKV replication effect was further investigated using the HMGB1-knockdown cells, shHMGB1 cells. shHMGB1 Huh7 cells were prepared by transfecting the recombinant lentivirus into wild-type (WT) Huh7 cells to achieve the stable knockdown of HMGB1 gene expression. Immunoblot analysis showed that the HMGB1-knockdown efficiency in shHMGB1 cells was 94% (Supplementary Fig. S1), and ELISA results showed a decrease in HMGB1 secretion in shHMGB1 cells for both the mock- (38 pg/mL) and dexamethasone-treated groups (0 pg/mL) groups (data not shown because the values were beyond the ELISA kit’s detection limit). Both shHMGB1 cells and WT Huh7 cells were infected with ZIKV at MOI of 1, followed by treatment with 150 µM of dexamethasone. The ZIKV replicated to a significantly higher titer (P < 0.05) in the shHMGB1 Huh 7 cells than in the WT Huh7 cells, with or without dexamethasone treatment (Fig. 7). Dexamethasone treatment of the infected shHMGB1 cells and WT Huh7 cells showed a reduction of 36% (P > 0.05) and 75% of ZIKV titer (P < 0.05), respectively, in comparison to the virus titers of the mock-treated infected cells (Fig. 7). This suggested that the HMGB1 could play a role in the antiviral actions of dexamethasone.

## Discussion

**Figure 8:**
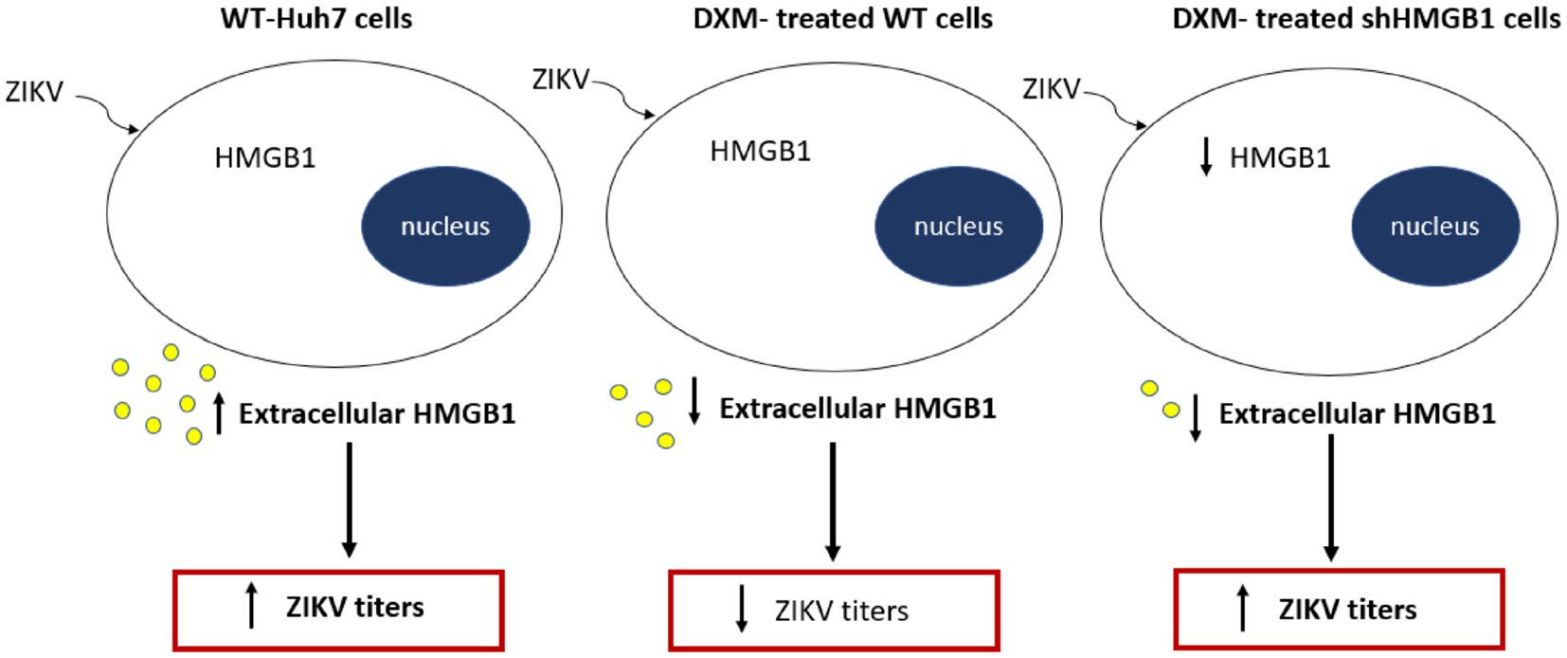
Model of HMGB1 and its role in ZIKV infection. ZIKV infection induces the translocation and release of HMGB1 in WT Huh7 cells. Treatment of dexamethasone in the ZIKV-infected WT cells inhibits the release of extracellular HMGB1 and ZIKV replication. However, the treatment in shHMGB1 cells does not cause the reduction of ZIKV titers, indicating the importance of the intracellular HMGB1 in ZIKV infection.

Findings from the present study demonstrated that the liver originated Huh7 cells were highly susceptible to ZIKV infection, and this finding was consistent with those reported in the earlier study^34^. In the study, it was demonstrated that the ZIKV infection induced translocation of HMGB1 from the nucleus to the cytoplasm and subsequently released extracellularly from the infected Huh7 cells. Treatment of Huh7 cells with dexamethasone, a well-known anti-inflammatory drug, significantly reduced ZIKV infection and the extracellular HMGB1 levels. In the HMGB1 knockdown Huh7 cells, however, the treatment with dexamethasone did not cause significant inhibition of ZIKV replication, suggesting the involvement of HMGB1 in regulating the antiviral mechanisms of dexamethasone (Fig. 8).

HMGB1 is a nuclear transcription factor that acts as a pro-inflammatory cytokine when released from the cells in response to infection, cell injury and inflammation^13,35^. In normal circumstances, the HMGB1 accumulates in the nucleus, giving a nuclear-to-cytoplasmic HMGB1 ratio of approximately 30:1^36^. During infection or cell injury, HMGB1 undergoes post-translational modifications, including acetylation, methylation and phosphorylation, causing its translocation from the nucleus to cytoplasm before being released into the extracellular environment^37,38^. For instance, the host cell p300/CBP-associated factor (PCAF) acetylase complex, triggered by dengue virus capsid protein, has been demonstrated to enhance the HMGB1 release^39^. Moreover, excessive production of reactive oxygen species (ROS) induced by respiratory syncytial virus and porcine circovirus-2 have been found to promote the release of HMGB1^40,41^. Similarly, our study showed that ZIKV infection could induce HMGB1 nuclear-to-cytoplasmic translocation and release in a time-dependent manner, most likely through the similar mechanisms described. Consistent with previous findings^42^, ZIKV-induced CPE (cytopathic effect) appeared at 72 h.p.i. in the infected Huh7 cells with MOI 1 (data not shown). Meanwhile, HMGB1 translocation was observed in the early time point of infection (24 h.p.i.) prior to the onset of ZIKV CPE (Fig. 2), suggesting that HMGB1 release might not be a sole secondary effect of ZIKV-induced CPE. In contrast, a prior study found that HIV-induced CPE was associated with the release of HMGB1, lactate dehydrogenase (LDH) activity and caspase-3 (c3) activation^14^. Future research is required to determine the HMGB1 response to ZIKV CPE, as well as the virus-induced necrosis or apoptosis. On the other hand, augmented serum levels of HMGB1 was observed in secondary dengue virus infection^22^, which has been associated with higher viral load and prolonged viremia, most probably due to the delayed virus clearance as reported earlier^43^. Meanwhile, our study also revealed that the secreted HMGB1 was elevated in an MOI-dependent manner.

Extracellular HMGB1 induces innate immune response via interaction with the receptor for advanced glycation end products (RAGE) or toll-like receptor (TLR-2 and -4), activating MyD88 and NF-κB signaling pathways, which then leads to the production of pro-inflammatory cytokines for virus clearance^44^. Additional research is needed to elucidate the potential protective role of extracellular HMGB1 on neighboring cells during ZIKV infection. However, excessive extracellular HMGB1 during virus infection has been associated with the pathogenesis of diseases^45–47^. For instance, extracellular HMGB1 was related to the pathogenesis of dengue hemorrhagic fever-dengue shock syndrome (DHF/DSS), presumably through the vascular barrier disruption^39^. Besides, extracellular HMGB1 has been correlated to neuroinvasion of ZIKV and Japanese encephalitis virus (JEV), possibly via disruption of the blood–brain barrier^24,48^. Therefore, preventing the overproduction of extracellular HMGB1 could be a therapeutic approach against the pathogenesis of viral infection.

Many studies have demonstrated that intracellular and extracellular HMGB1 play different roles in virus replication. For example, intracellular HMGB1 facilitates influenza virus and hepatitis C virus replication by directly interacting with viral RNA or nucleoprotein^49,50^. Vice versa, intracellular HMGB1 limits HIV replication by downregulating the long terminal repeat (LTR)-mediated transcription^51^. Extracellular HMGB1 has also been reported to promote HIV replication in monocytic cells but decreases viral replication in primary macrophages^52^. Moreover, the antiviral effect of extracellular HMGB1 against the hepatitis C virus has been established by the activation of the interferon signaling in virus-infected human hepatoma cell line^15^. Although HMGB1 has been found to have significant effects on various virus replication, the role of HMGB1 in ZIKV infection remains largely unknown.

Dexamethasone’s anti-inflammatory properties have been widely used in clinical trials to protect against liver failure, with doses ranging from 10 to 25 mg/day (25.5–63.7 µM)^53–55^. However, high doses of dexamethasone, 40–100 mg/day (102–254.8 µM) have also been used in previous clinical studies to treat other diseases^56–58^. Therefore, the dexamethasone doses used in this study (5, 80 and 150 µM) are clinically achievable and safe for humans. This study revealed that dexamethasone treatment decreased both extracellular HMGB1 release and ZIKV replication. Dexamethasone has been shown to inhibit HMGB1 nuclear-to-cytoplasmic translocation via acetyltransferase attenuation, hence reducing the HMGB1-TLR4 inflammatory pathway^29^. A previous study also found that dexamethasone treatment does not significantly alter intracellular HMGB1 protein expression^59^. As a result, it is suggested that dexamethasone exerts an effect on HMGB1 translocation rather than transcription and translation of the gene. Moreover, dexamethasone’s anti-inflammatory action, which reduces TNF-a, IFN-a, and IL-10 production, has also been demonstrated to reduce dengue virus replication^32^. Therefore, the inhibition of ZIKV replication by dexamethasone treatment was likely through the prevention of HMGB1 extracellular release.

To further investigate the role of intracellular HMGB1 in the antiviral mechanism of dexamethasone against ZIKV infection, HMGB1-knockdown Huh7 cells were used. The Huh7 cell line is fortunately a reliable cell line for the gene knockdown study as stable transfection and/or higher viral transduction efficiency can be achieved^60,61^. This study showed no antiviral effects in dexamethasone-treated HMGB1-knockdown cells, signifying that the presence of intracellular HMGB1 is critical for the dexamethasone’s antiviral activity. This finding is consistent with several earlier studies which demonstrated that HMGB1 retained in the nucleus plays a role in the antiviral response against dengue virus^62^, duck reovirus, duck Tembusu, duck plague virus^63^ and human immunodeficiency virus^51^. In addition, the role of nuclear HMBG1 retention in upregulating interferon-stimulated genes (ISGs) expression to antagonize virus replication has been reported^62,64^. Thus, the anti-ZIKV mechanism of dexamethasone is most likely through preventing HMGB1 translocation, which results in HMGB1 retention in the cells. Because HMGB1 is a multifunctional protein with a range of activities depending on its location, blocking HMGB1 translocation could have unintended consequences such as an imbalance of mitochondrial homeostasis and autophagy machinery. Dexamethasone has been shown to inhibit HMGB1 nuclear-to-cytoplasmic translocation via acetyltransferase attenuation^29^. However, completely blocking HMGB1 translocation by dexamethasone is unlikely to happen as multiple post-translational modifications such as acetylation^65^, methylation^38^ and phosphorylation^66^ are involved in the active secretion of HMGB1 from the nucleus to the cytosol compartment.

In conclusion, current findings suggest that ZIKV infection triggers HMGB1 translocation from the nucleus to the cytoplasm and extracellular release in a time- and MOI-dependent manner. The findings of this study provide a better insight into the underlying anti-ZIKV mechanisms by dexamethasone, particularly through the regulation of HMGB1. While the current observations are based on the study on a single ZIKV strain in a single cell line, additional studies using other ZIKV strains and other cell lines or primary cells are desired to rule out the possible cell- or virus-dependent effects. Further in vivo investigation is also important to provide a better understanding of HMGB1’s role in ZIKV infection.

## Methods

### Cell culture and virus

Human hepatocellular carcinoma cells (Huh7,1 JCRB 0403) were cultured in Roswell Park Memorial Institute 1640 medium (RPMI; RBRC-RCB1942), enriched with 10% of heat-inactivated fetal bovine serum (FBS; Bovogen, Australia) and L-glutamine (Gibco, NY, USA). Huh7 cells were incubated at 37 °C in a humidified incubator with 5% CO_2_. Asian Zika virus (ZIKV) strain P6-740 (from Malaysia) was used in this study as it has been extensively used in in vitro antiviral studies^67–69^ and in vivo mouse models, implying that it is more pathogenic than other Asian ZIKV strains (PRVABC59 and FSS 13025)^70^. ZIKV was archived in Tropical Infectious Diseases Research & Education Centre (TIDREC) at the University of Malaya, Kuala Lumpur, Malaysia. African Green monkey kidney cells (Vero, ATCC-CCL81) in Dulbecco’s Modified Eagle Medium (DMEM; Gibco, NY, USA) were used for virus propagation and titration.

### Virus infection and virus titer determination

Huh7 cells were infected with ZIKV at various MOIs, with and without dexamethasone (Sigma-Aldrich, St. Louis, MO, USA) added to cells at different concentrations. The cell culture supernatants were then collected at different time points (e.g., 24 h, 48 h and 72 h) for ZIKV titer determination using the focus-forming assay and expressed as Focus-Forming-Unit per ml (FFU/mL). The assay was carried out as previously reported^33^.

### Enzyme-linked immunosorbent assay (ELISA)

The amount of extracellular HMGB1 in the cell culture supernatant was quantified using a commercially available human HMGB1 ELISA kit (Cloud-Clone Corp, USA; cat. SEA399Hu 96 tests) according to the manufacturer’s instructions. The detection limit of the ELISA kit is 62.4 pg/mL.

### Immunoblot assay

Huh7 cells were harvested at 24, 48 and 72 h after ZIKV infection. Proteins were extracted from Huh7 cells as directed by the manufacturer using the NE-PER cytoplasmic and nuclear protein extraction kit (Thermo Scientific Pierce, Rockford, IL, USA). Micro BCA Protein Assay Kit (Thermo Scientific Pierce, Rockford, IL, USA) was used to quantify the protein concentration of each sample. Equal amounts of proteins were denatured with sodium dodecyl sulphate–sample loading buffer, separated using sodium dodecyl sulphate–polyacrylamide gel electrophoresis, and transferred to a polyvinylidene fluoride membrane for 14 min by the Bio-Rad semi-dry transfer system (Hercules, CA). Afterward, the membranes were blocked with 3% skimmed milk in PBS for 1 h at room temperature before being incubated with primary antibodies overnight at 4 °C. The primary antibodies used in this study were as follows: rabbit anti-HMGB1 antibody (ab79823; 1: 1:10,000–1:50,000 dilution; Abcam, Cambridge, UK), rabbit anti-GADPH antibody (ab9485, 1:2500; Abcam, Cambridge, UK) and rabbit anti-PARP1 antibody (PLA0184, 1:2000–1:10,000 dilution, Sigma-Aldrich, St. Louis, MO, USA). Blots were then incubated for 1 h at room temperature with a secondary goat anti-rabbit IgG antibody conjugated with horseradish peroxidase (HRP) (65–6120, 1:3000–1:10,000; Invitrogen, Carlsbad, CA, USA). The chemiluminescence Western blotting kit (BioRad, Hercules, CA) was used to detect the presence of proteins.

### HMGB1 gene knockdown in Huh7 cells

The shRNA approach was used to achieve a stable knockdown of HMGB1 gene expression (shHMGB1)^71^. The recombinant lentiviruses carrying shRNA plasmid targeting human HMGB1 gene were purchased from OriGene Technologies (TL316576V, Rockville, MD, USA). A 50 percent confluent Huh7 cell monolayer was infected with the recombinant lentiviruses at an MOI of 20 in the presence of 8 µg/mL of polybrene, which was then incubated at 37 °C overnight. After 24 h, the culture medium was changed to a puromycin-containing selective medium with a final concentration of 1 µg/mL (Sigma-Aldrich, St. Louis, MO).

### Statistical analysis

The statistically significant differences between the cell groups were determined by employing a one-way analysis of variance (ANOVA) and Bonferroni’s multiple-comparison test with a 95 percent confidence interval (C.I.) to determine the significance of the variance. Graph-Pad Prism 8 was used to perform all statistical tests. The statistical significances of the groups were as follows: *P < 0.05, **P < 0.01, ***P < 0.001.

## Supplementary Information

Supplementary Figure S1.

Supplementary Figure S2.

Supplementary Figure S3.

## References

[1] Lanciotti RS, Lambert AJ, Holodniy M, Saavedra S, Signor LDCC (2016). Phylogeny of Zika virus in western hemisphere, 2015. Emerg. Infect. Dis..

[2] Sikka V (2016). The emergence of Zika virus as a global health security threat: A review and a consensus statement of the INDUSEM Joint Working Group (JWG). J. Glob. Infect. Dis..

[3] Schuler-Faccini L (2016). Possible association between Zika virus infection and microcephaly—Brazil, 2015. Morb. Mortal. Wkly. Rep..

[4] Dominguez-Moreno R (2014). Mortality associated with a diagnosis of Guillain-Barré syndrome in adults of Mexican health institutions. Rev. Neurol..

[5] Jampol LM, Goldstein DA (2016). Zika virus infection and the eye. JAMA Ophthalmol..

[6] Coffey LL (2017). Zika virus tissue and blood compartmentalization in acute infection of rhesus macaques. PLoS ONE.

[7] Gourinat A-C, O’Connor O, Calvez E, Goarant C, Dupont-Rouzeyrol M (2015). Detection of Zika virus in urine. Emerg. Infect. Dis..

[8] Hirsch AJ (2017). Zika Virus infection of rhesus macaques leads to viral persistence in multiple tissues. PLoS Pathog..

[9] Sherman KE (2019). Zika virus replication and cytopathic effects in liver cells. PLoS ONE.

[10] Bandeira AC (2020). Clinical and laboratory findings of acute Zika virus infection in patients from Salvador during the first Brazilian epidemic. Braz. J. Infect. Dis..

[11] Macnamara F (1954). Zika virus: A report on three cases of human infection during an epidemic of jaundice in Nigeria. Trans. R. Soc. Trop. Med. Hyg..

[12] Lange SS, Mitchell DL, Vasquez KM (2008). High mobility group protein B1 enhances DNA repair and chromatin modification after DNA damage. Proc. Natl. Acad. Sci..

[13] Yang H, Wang H, Czura CJ, Tracey KJ (2005). The cytokine activity of HMGB1. J. Leukoc. Biol..

[14] Barqasho B, Nowak P, Abdurahman S, Walther-Jallow L, Sönnerborg A (2010). Implications of the release of high-mobility group box 1 protein from dying cells during human immunodeficiency virus type 1 infection in vitro. J. Gen. Virol..

[15] Jung JH (2011). Hepatitis C virus infection is blocked by HMGB1 released from virus-infected cells. J. Virol..

[16] Kamau E (2009). Dengue virus infection promotes translocation of high mobility group box 1 protein from the nucleus to the cytosol in dendritic cells, upregulates cytokine production and modulates virus replication. J. Gen. Virol..

[17] Gougeon M, Melki M, Saidi H (2012). HMGB1, an alarmin promoting HIV dissemination and latency in dendritic cells. Cell Death Differ..

[18] Chu J, Ng M (2003). The mechanism of cell death during West Nile virus infection is dependent on initial infectious dose. J. Gen. Virol..

[19] Borde C (2011). Stepwise release of biologically active HMGB1 during HSV-2 infection. PLoS ONE.

[20] Chen R (2020). HMGB1 as a potential biomarker and therapeutic target for severe COVID-19. Heliyon.

[21] Liu Y (2012). Hepatitis B virus X protein stabilizes amplified in breast cancer 1 protein and cooperates with it to promote human hepatocellular carcinoma cell invasiveness. Hepatology.

[22] Allonso D (2012). Elevated serum levels of high mobility group box 1 (HMGB1) protein in dengue-infected patients are associated with disease symptoms and secondary infection. J. Clin. Virol..

[23] Rashid M-U (2020). Zika virus dysregulates human Sertoli cell proteins involved in spermatogenesis with little effect on tight junctions. PLoS Negl. Trop. Dis..

[24] de Carvalho GC (2019). RAGE and CCR7 mediate the transmigration of Zika-infected monocytes through the blood-brain barrier. Immunobiology.

[25] Fares-Gusmao R (2019). Differential pattern of soluble immune markers in asymptomatic dengue, West Nile and Zika Virus Infections. Sci. Rep..

[26] Xu L, Bao H, Si Y, Wang X (2013). Effects of dexmedetomidine on early and late cytokines during polymicrobial sepsis in mice. Inflamm. Res..

[27] Zhao S (2018). Dexamethasone inhibits NF-кBp65 and HMGB1 expression in the pancreas of rats with severe acute pancreatitis. Mol. Med. Rep..

[28] Liu X (2018). Paeonol attenuates acute lung injury by inhibiting HMGB1 in lipopolysaccharide-induced shock rats. Int. Immunopharmacol..

[29] Zhang J (2016). HMGB1-TLR4 signaling participates in renal ischemia reperfusion injury and could be attenuated by dexamethasone-mediated inhibition of the ERK/NF-κB pathway. Am. J. Transl. Res..

[30] Lee J-S, Kim S-R, Song J-H, Lee Y-P, Ko H-J (2018). Anti-human rhinovirus 1B activity of dexamethasone viaGCR-dependent autophagy activation. Osong Public Health Res. Perspect..

[31] Ilott M, Salt J, Gaskell R, Kitching R (1997). Dexamethasone inhibits virus production and the secretory IgA response in oesophageal–pharyngeal fluid in cattle persistently infected with foot-and-mouth disease virus. Epidemiol. Infect..

[32] Reis SRNI (2007). An in vitro model for dengue virus infection that exhibits human monocyte infection, multiple cytokine production and dexamethasone immunomodulation. Mem. Inst. Oswaldo Cruz.

[33] Teoh B-T (2013). Dengue virus type 1 clade replacement in recurring homotypic outbreaks. BMC Evol. Biol..

[34] Chan JF-W (2016). Differential cell line susceptibility to the emerging Zika virus: Implications for disease pathogenesis, non-vector-borne human transmission and animal reservoirs. Emerg. Microbes Infect..

[35] Andersson U, Erlandsson-Harris H, Yang H, Tracey KJ (2002). HMGB1 as a DNA-binding cytokine. J. Leukoc. Biol..

[36] Kuehl L, Salmond B, Tran L (1984). Concentrations of high-mobility-group proteins in the nucleus and cytoplasm of several rat tissues. J. Cell Biol..

[37] Tang D (2010). HMGB1 release and redox regulates autophagy and apoptosis in cancer cells. Oncogene.

[38] Ito I, Fukazawa J, Yoshida M (2007). Post-translational methylation of high mobility group box 1 (HMGB1) causes its cytoplasmic localization in neutrophils. J. Biol. Chem..

[39] Ong SP, Lee LM, Leong YFI, Ng ML, Chu JJH (2012). Dengue virus infection mediates HMGB1 release from monocytes involving PCAF acetylase complex and induces vascular leakage in endothelial cells. PLoS ONE.

[40] Hosakote YM, Brasier AR, Casola A, Garofalo RP, Kurosky A (2016). Respiratory syncytial virus infection triggers epithelial HMGB1 release as a damage-associated molecular pattern promoting a monocytic inflammatory response. J. Virol..

[41] Sun R (2020). PCV2 induces reactive oxygen species to promote nucleocytoplasmic translocation of the viral DNA binding protein HMGB1 to enhance its replication. J. Virol..

[42] Van der Hoek KH (2017). Viperin is an important host restriction factor in control of Zika virus infection. Sci. Rep..

[43] Wang W-K (2006). Slower rates of clearance of viral load and virus-containing immune complexes in patients with dengue hemorrhagic fever. Clin. Infect. Dis..

[44] Lotze MT, Tracey KJ (2005). High-mobility group box 1 protein (HMGB1): Nuclear weapon in the immune arsenal. Nat. Rev. Immunol..

[45] Qu Y (2018). Newcastle disease virus infection triggers HMGB1 release to promote the inflammatory response. Virology.

[46] Duan E (2014). Porcine reproductive and respiratory syndrome virus infection triggers HMGB1 release to promote inflammatory cytokine production. Virology.

[47] Chen S (2017). Hepatitis B virus X protein stimulates high mobility group box 1 secretion and enhances hepatocellular carcinoma metastasis. Cancer Lett..

[48] Zou, S.-S. *et al.* Brain microvascular endothelial cells-derived HMGB1 facilitates monocyte transendothelial migration favoring JEV neuroinvasion (2020).

[49] Moisy D (2012). HMGB1 protein binds to influenza virus nucleoprotein and promotes viral replication. J. Virol..

[50] Yu R (2015). HMGB1 promotes hepatitis C virus replication by interaction with stem-loop 4 in the viral 5′ untranslated region. J. Virol..

[51] Naghavi MH (2003). Intracellular high mobility group B1 protein (HMGB1) represses HIV-1 LTR-directed transcription in a promoter-and cell-specific manner. Virology.

[52] Nowak P (2006). HMGB1 activates replication of latent HIV-1 in a monocytic cell-line, but inhibits HIV-1 replication in primary macrophages. Cytokine.

[53] Zhang XQ (2011). Efficacy of short-term dexamethasone therapy in acute-on-chronic pre-liver failure. Hepatol. Res..

[54] Kemeny N (1994). Phase II study of hepatic arterial floxuridine, leucovorin, and dexamethasone for unresectable liver metastases from colorectal carcinoma. J. Clin. Oncol..

[55] Kemeny NE (2011). Treating primary liver cancer with hepatic arterial infusion of floxuridine and dexamethasone: Does the addition of systemic bevacizumab improve results. Oncology.

[56] San Miguel JF (2007). Dexamethasone dose adjustments seem to result in better efficacy and improved tolerability in patients with relapsed/refractory multiple myeloma who are treated with lenalidomide/dexamethasone (MM009/010 sub-analysis). Blood.

[57] Sørensen P, Helweg-Larsen S, Mouridsen H, Hansen H (1994). Effect of high-dose dexamethasone in carcinomatous metastatic spinal cord compression treated with radiotherapy: A randomised trial. Eur. J. Cancer.

[58] Cheng Y (2003). Initial treatment of immune thrombocytopenic purpura with high-dose dexamethasone. N. Engl. J. Med..

[59] Hou C (2010). Expression of high mobility group box-1 in the lung tissue and BALF of asthmatic mice and the influence of dexamethasone. J. South. Med. Univ..

[60] Chong ZX, Yeap SK, Ho WY (2021). Transfection types, methods and strategies: A technical review. PeerJ.

[61] Condreay JP, Witherspoon SM, Clay WC, Kost TA (1999). Transient and stable gene expression in mammalian cells transduced with a recombinant baculovirus vector. Proc. Natl. Acad. Sci..

[62] Zainal N (2017). Resveratrol treatment reveals a novel role for HMGB1 in regulation of the type 1 interferon response in dengue virus infection. Sci. Rep..

[63] Hou X (2020). High-mobility group box 1 protein (HMGB1) from Cherry Valley duck mediates signaling pathways and antiviral activity. Vet. Res..

[64] Ma C, Wang Y, Dong L, Li M, Cai W (2015). Anti-inflammatory effect of resveratrol through the suppression of NF-κB and JAK/STAT signaling pathways. Acta Biochim. Biophys. Sin..

[65] Bonaldi T (2003). Monocytic cells hyperacetylate chromatin protein HMGB1 to redirect it towards secretion. EMBO J..

[66] Youn JH, Shin J-S (2006). Nucleocytoplasmic shuttling of HMGB1 is regulated by phosphorylation that redirects it toward secretion. J. Immunol..

[67] Kim J, Seong R-K, Kumar M, Shin OS (2018). Favipiravir and ribavirin inhibit replication of Asian and African strains of Zika virus in different cell models. Viruses.

[68] Mohd A, Zainal N, Tan K-K, AbuBakar S (2019). Resveratrol affects Zika virus replication in vitro. Sci. Rep..

[69] Snyder B, Goebel S, Koide F, Ptak R, Kalkeri R (2018). Synergistic antiviral activity of Sofosbuvir and type-I interferons (α and β) against Zika virus. J. Med. Virol..

[70] McDonald EM (2019). Duration of seminal Zika viral RNA shedding in immunocompetent mice inoculated with Asian and African genotype viruses. Virology.

[71] Hashemi M, Zali A, Hashemi J, Oraee-Yazdani S, Akbari A (2018). Down-regulation of 14-3-3 zeta sensitizes human glioblastoma cells to apoptosis induction. Apoptosis.

