# Supplementary material for: Intracellular translocation of HMGB1 is important for Zika virus replication in Huh7 cells
Source: Sci Rep. 2022 Jan 20;12:1054. doi: 10.1038/s41598-022-04955-z (PMC8776752; doi:10.1038/s41598-022-04955-z)
Supplement: Supplementary file 1 — Supplementary Figure S1. [file 41598_2022_4955_MOESM1_ESM.docx]

# Supplementary Figure Legends

**Supplementary figure S1. Confirmation of HMGB1-knockdown by immunoblot.** Cell lysates of wild-type (WT) and HMGB1-knockdown (shHMGB1) Huh7 cells were harvested and subjected to immunoblot for HMGB1 detection.


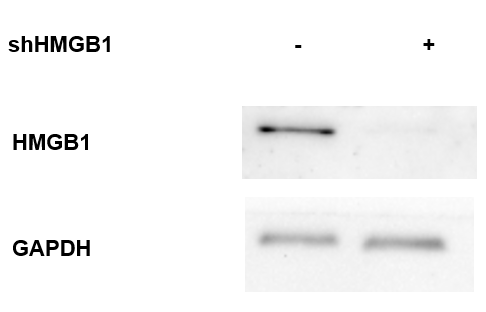


**Supplementary figure S1. Confirmation of HMGB1-knockdown by immunoblot.**
