# Supplementary material for: Intracellular translocation of HMGB1 is important for Zika virus replication in Huh7 cells
Source: Sci Rep. 2022 Jan 20;12:1054. doi: 10.1038/s41598-022-04955-z (PMC8776752; doi:10.1038/s41598-022-04955-z)
Supplement: Supplementary file 2 — Supplementary Figure S2. [file 41598_2022_4955_MOESM2_ESM.docx]

# Supplementary Figure Legends

# Supplementary figure S2. Full-length blots of Figure 2a and b.

|  | **Cytoplasm** | | | |  | **Nucleus** | | | |
| --- | --- | --- | --- | --- | --- | --- | --- | --- | --- |
| **ZIKV (MOI=1)** | **-** | **+** | **+** | **+** |  | **-** | **+** | **+** | **+** |
| **Hour post infection (h.p.i)** | **-** | **24** | **48** | **72** |  | **-** | **24** | **48** | **72** |


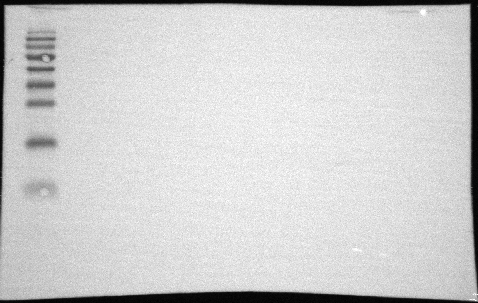

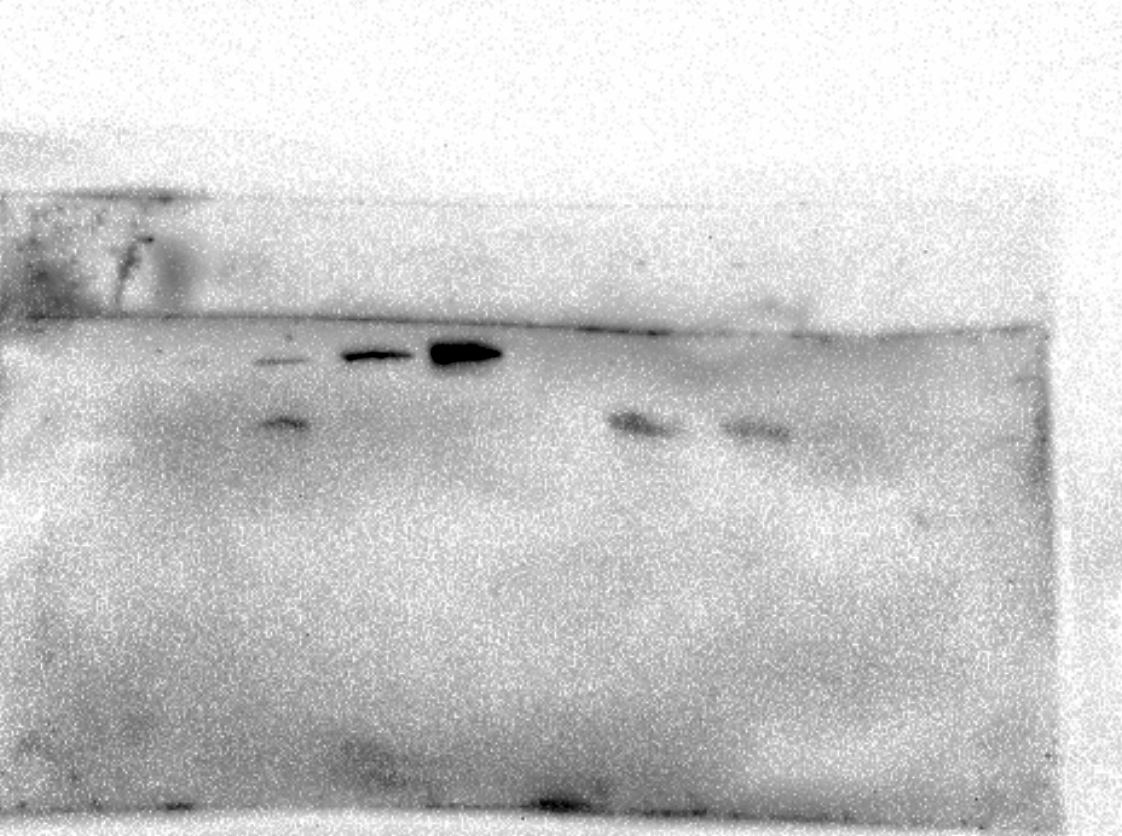


**HMGB1**

**25, 30kDa**

34 kDa

26 kDa

* The ladder used in this experiment was not chemiluminescence-based. Thus, the ladder image was not captured alongside the blots. The molecular mass of nuclear HMGB1 was ~25kDa, whereas the molecular mass of cytoplasm HMGB1 was~ 30 kDa, most likely due to acetylation.

**Supplementary figure S2. Full-length blots of Figure 2a and b.**

|  | **Nucleus** | | | |  | **Cytoplasm** | | | |
| --- | --- | --- | --- | --- | --- | --- | --- | --- | --- |
| **ZIKV (MOI=1)** | **-** | **+** | **+** | **+** |  | **-** | **+** | **+** | **+** |
| **Hour post infection (h.p.i)** | **-** | **24** | **48** | **72** |  | **-** | **24** | **48** | **72** |


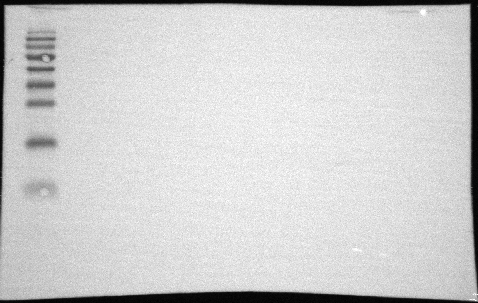

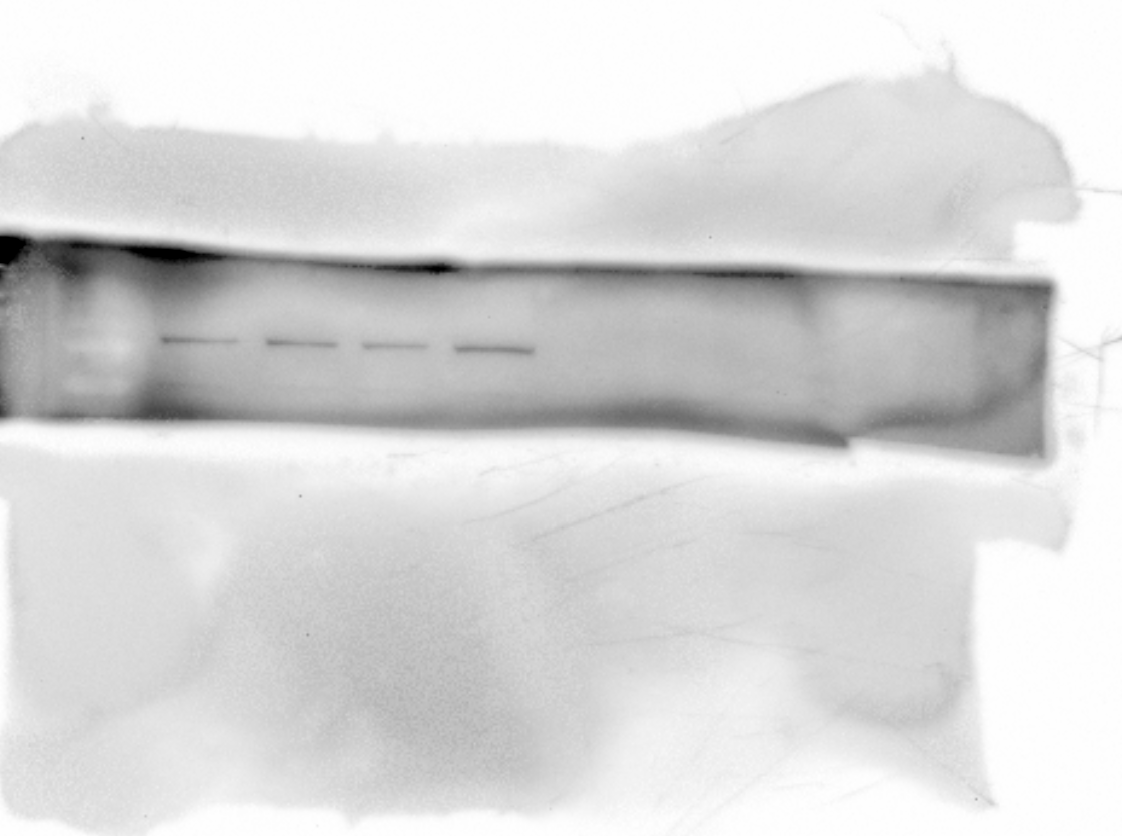


**PARP-1**

**117 kDa**

130 kDa

95 kDa


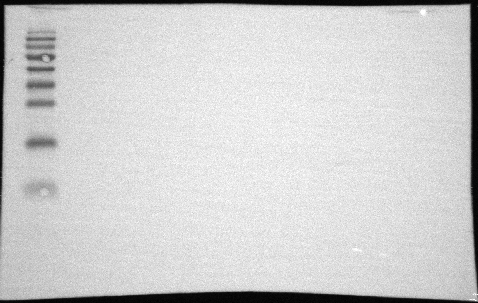

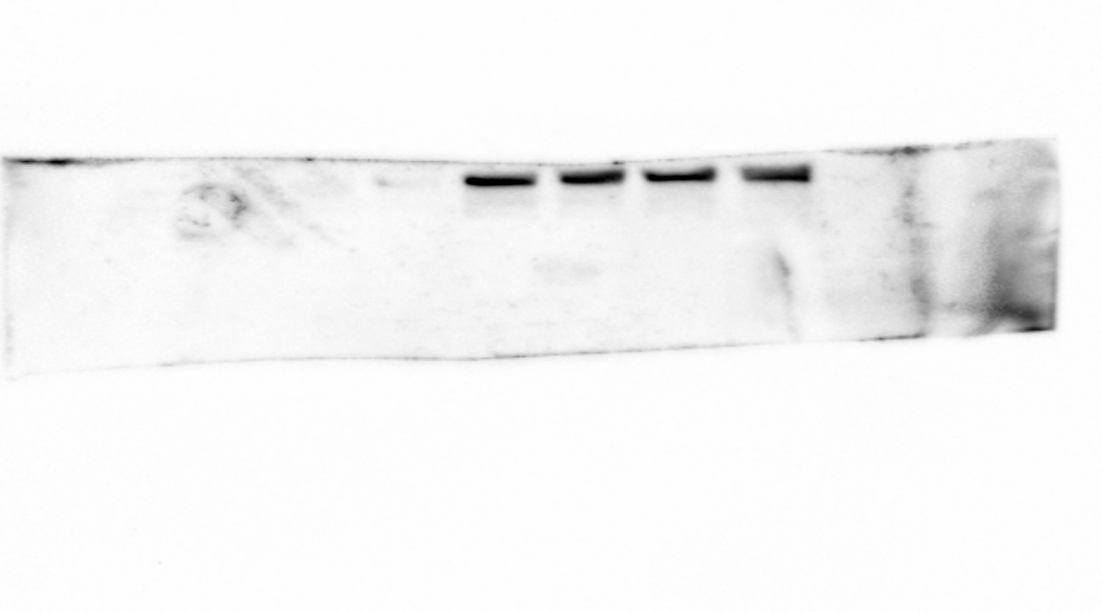


34 kDa

**GAPDH**

**37 kDa**

**Supplementary figure S2. Full-length blots of Figure 2a and b.**
