# Supplementary material for: Intracellular translocation of HMGB1 is important for Zika virus replication in Huh7 cells
Source: Sci Rep. 2022 Jan 20;12:1054. doi: 10.1038/s41598-022-04955-z (PMC8776752; doi:10.1038/s41598-022-04955-z)
Supplement: Supplementary file 3 — Supplementary Figure S3. [file 41598_2022_4955_MOESM3_ESM.docx]

# Supplementary Figure Legends

# Supplementary figure S3. Full-length blots of Supplementary figure S1

| **shHMGB1** | **-** | **+** |
| --- | --- | --- |

**HMGB1**

**25 kDa**


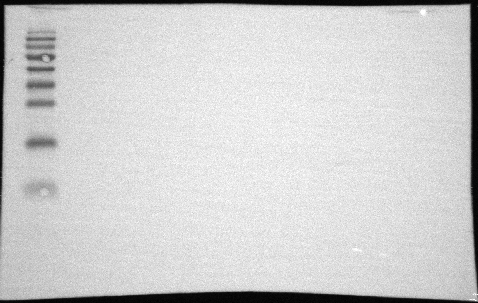

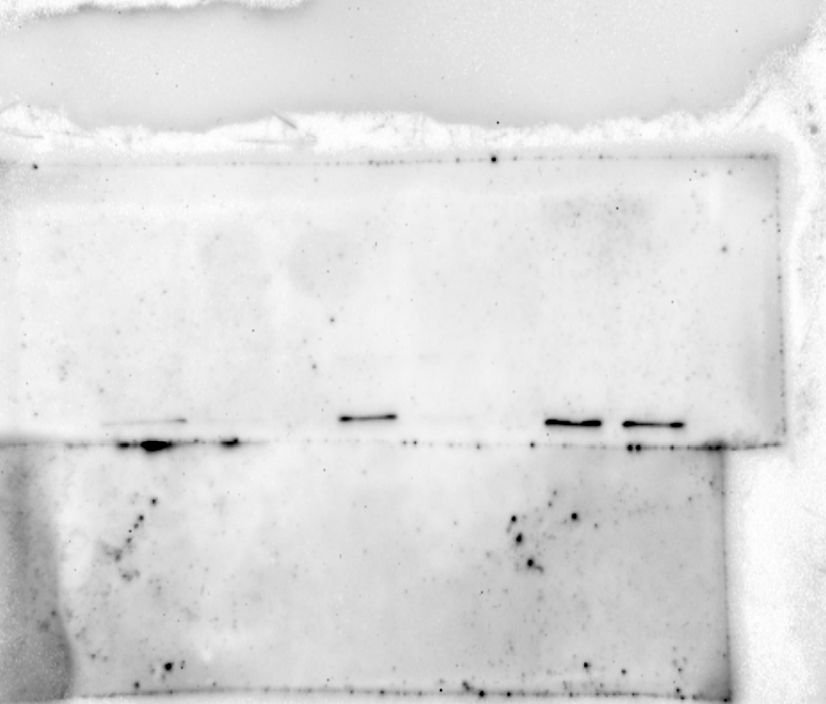


26 kDa


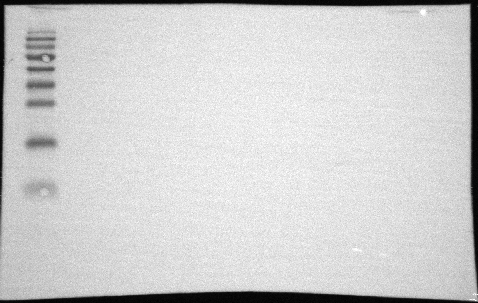

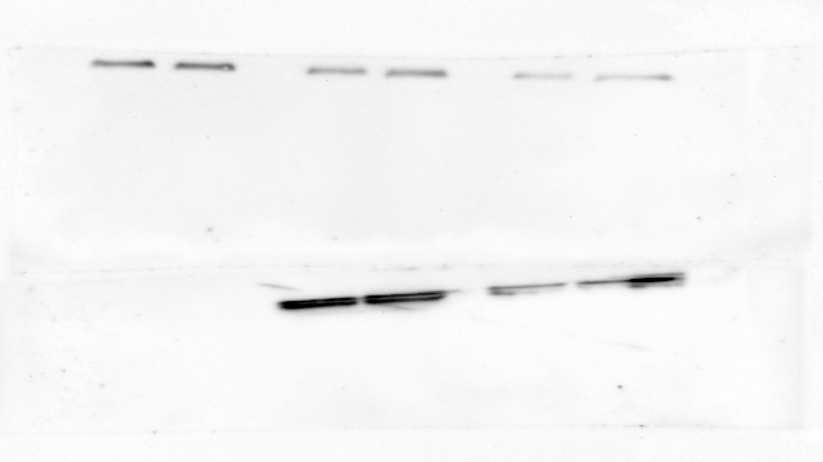


**GAPDH**

**37 kDa**

34 kDa

*HMGB1-knockdown cells were used to investigate the role of HMGB1 in ZIKV replication. Western blot analysis showed that the HMGB1-knockdown (shHMGB1) Huh7 cells infected with shRNA B (middle) lentivirus had the highest inhibition level of HMGB1 expression. Thus, the shHMGB1 B cells were used for the following experiments.

# Supplementary figure S3. Full-length blots of Supplementary figure S1
